# Plasma Circular RNAs hsa_circ_0001953 and hsa_circ_0009024 as Diagnostic Biomarkers for Active Tuberculosis

**DOI:** 10.3389/fmicb.2018.02010

**Published:** 2018-08-30

**Authors:** Zikun Huang, Rigu Su, Cheng Qing, Yiping Peng, Qing Luo, Junming Li

**Affiliations:** ^1^Department of Clinical Laboratory, The First Affiliated Hospital of Nanchang University, Nanchang, China; ^2^Intensive Care Unit, The First Affiliated Hospital of Nanchang University, Nanchang, China; ^3^Department of Tuberculosis, Jiangxi Chest Hospital, Nanchang, China

**Keywords:** tuberculosis, circular RNAs, plasma, transcriptome, biomarkers

## Abstract

Recent studies have demonstrated that circular RNAs (circRNAs) could serve as potential molecular markers for disease diagnosis; however, little is known about their diagnostic value in active tuberculosis (TB). This study first performed a microarray screening of circRNA changes in plasma samples from 3 patients with active pulmonary TB and 3 healthy controls. Then, candidate circRNAs were selected for validation on a quantitative real-time PCR system. Of the 61 differentially expressed circRNAs recorded, 43 and 18 were upregulated and downregulated in the TB group, respectively. Validation assays demonstrated that plasma levels of 6 circRNAs, including hsa_circ_0009024, hsa_circ_0001953, hsa_circ_0008297, hsa_circ_0003528, hsa_circ_0003524 and hsa_circ_0015879 were remarkably increased in TB patients. Plasma levels of hsa_circ_0001953 and hsa_circ_0009024 were correlated with TB severity. Next, hsa_circ_0001953 and hsa_circ_0009024 were assessed in an independent cohort consisting of 120 TB patients and 100 control individuals. An area under the receiver operating characteristic (ROC) curve of 0.915 (95% confidence interval 0.880-0.951; *P* < 0.001) was obtained for detecting TB, with hsa_circ_0001953 and hsa_circ_0009024 used in combination. Additionally, plasma levels of hsa_circ_0001953 and hsa_circ_0009024 were reduced significantly in patients after treatment (*P* < 0.001). The present findings indicate that the circRNAs hsa_circ_0001953 and hsa_circ_0009024 may represent novel plasma biomarkers for active TB diagnosis.

## Introduction

Tuberculosis (TB) is the ninth leading cause of death worldwide and the leading cause from a single infectious agent, with 1.3 million deaths and 10.4 million new cases worldwide in 2016 (WHO, 2017). Accurate and early diagnosis is important for controlling infection and effective treatment of TB. The current conventional methods for TB diagnosis are primarily smears for acid-fast bacilli (AFB) and *Mycobacterium tuberculosis* culture (Kranzer et al., 2016). However, the AFB smear positivity rate among TB cases was found to be only 20–30%, and the culture requires 4–8 weeks for the growth of *M. tuberculosis* (Dheda et al., 2016). New automatic molecular methods, such as GeneXpert MTB/RIF for the diagnosis of TB, are currently available; however, the cost is too high for resource-limited settings without committed long-term, external funding (Albert et al., 2016; García-Basteiro et al., 2016). Therefore, identifying appropriate molecular biomarkers for early diagnosis of TB is in urgent need.

Circular RNAs (circRNAs) are a novel class of RNAs that participate in several physiological and pathological processes (Liu et al., 2017; Ng et al., 2017). Growing evidence has suggested that many circRNAs can function as competing endogenous (ceRNAs) RNAs to block the functions of their target microRNAs (miRNAs) by binding target miRNAs, a mechanism called miRNA sponging (Kulcheski et al., 2016; Rong et al., 2017). Different from linear RNAs, circRNAs form a circular structure closed by covalent bonds, with high stability; therefore, many circRNAs can resist RNase digestion to survive in peripheral blood, interstitial fluid and saliva (Memczak et al., 2015). Recent researches have shown that circRNAs can serve as molecular biomarkers in the diagnosis and prognosis of many diseases. For example, Zhao et al. found that hsa_circ_0001275 in peripheral blood mononuclear cells (PBMCs) can be used to diagnose postmenopausal osteoporosis (Zhao et al., 2018), whereas Li et al. demonstrated that four plasma circRNAs (hsa_circ_102584, hsa_circ_400011, hsa_circ_101471 and hsa_circ_100226) can be used as diagnostic biomarkers of systemic lupus erythematosus (Li et al., 2018). Furthermore, Yin et al. reported that plasma hsa_circ_0001785 acts as a diagnostic biomarker for breast cancer detection (Yin et al., 2017). Despite increasing evidence that circRNAs widely participate in human diseases exist, the role of circRNAs in TB patients is largely unknown. Our previous study revealed some circRNAs were differentially expressed in the PBMCs between TB patients and healthy controls (Huang et al., 2018). In this study, the differentially expressed circRNAs in the plasma of TB patients were screened in a new cohort of subjects, the roles of plasma circulating circRNAs as novel biomarkers for active TB diagnosis were also investigated.

## Materials and methods

### Patients and plasma collection

Patients (*n* = 173) with active pulmonary TB were consecutively enrolled from the First Affiliated Hospital of Nanchang University and Jiangxi Chest Hospital, China, between May 2015 and January 2017. All TB cases were clinically diagnosed and confirmed as active pulmonary TB by positive AFB smear staining or sputum culture. The patients were then grouped by case severity, including minimal, moderate, and advanced disease stages based on chest radiology (Abakay et al., 2015). Each X-ray was scored independently by two different clinicians. In case of divergent opinions, the score was decided by both after discussion. If the disagreement persisted after discussion, a third senior clinician was invited for decision making. The subjects with other co-morbidities were excluded. Then, 25 hospitalized cases with active pulmonary TB were administered a 2HRZE/4HR regimen, starting with 2-month combinatory therapy with rifampicin (RMP, R), isoniazid (INH, H), pyrazinamide (PZA, Z) and ethambutol (EMB, E), followed by HR administration for 4 months. Then, the treated subjected were assessed based on symptoms, bacterial presence and radiological evaluation, and all 25 showed full recovery. Healthy controls (*n* = 153) seeking annual check-up, without clinical diagnosis of any infectious disease, diabetes and malignancy, and no close contact with tuberculosis patients, were randomly enrolled from outpatient clinics of the First Affiliated Hospital of Nanchang University (Nanchang, China). As diseased controls, 120 patients with lung ailments (40 pneumonia, 40 COPD, and 40 lung cancer cases), confirmed clinically after eliminating pulmonary TB, were enrolled from the First Affiliated Hospital of Nanchang University and Jiangxi Chest Hospital from July 2015 to December 2016. It should be noted that there are 52 TB patients, 21 healthy controls, 9 lung cancer patients, 7 pneumonia patients, and 10 COPD patients who enrolled in this study and overlap with the subjects of one of our previous study (Huang et al., 2018). All subjects with hypertension, diabetes, autoimmune diseases, a history of immunosuppressive drug use, hepatitis B, hepatitis C, or HIV infection were excluded from the analysis. This study had approval from by the Ethics Committee of the First Affiliated Hospital of Nanchang University; all participants provided signed informed consent. The protocols complied with the Declaration of Helsinki. Blood samples (5 mL) were collected from all subjects in K2-EDTA tubes and centrifuged at 2,000 g for 10 min at 4°C, followed by 12,000 g for 10 min at 4°C. The supernatants (plasma) were carefully collected and stored at −80°C until use. All specimens in this study were verified to have no hemolysis by appearance observation and determination of free hemoglobin (Hb) in plasma before the experiments. All experiments were carried out according to the Laboratory Biosafety Manual of the Clinical Laboratory of the First Affiliated Hospital of Nanchang University in a level II biological safety laboratory. With the exception of centrifugation, sample processing was performed under a level II biological safety cabinet. Before centrifugation, the samples were sealed in centrifuge tubes with screw caps.

### Total RNA isolation

Total RNA extraction from plasma specimens was carried out with miRNeasy Mini Kit (Qiagen, Germany). RNA integrity and quantity were assessed on a NanoDrop™1000 spectrophotometer (NanoDrop Technologies, USA).

### Microarray analysis of circRNA level assessment

Six RNA specimens were assessed by KANGCHEN (Shanghai, China) using Arraystar circRNA Microarray (Arraystar Inc., Rockville, MD, USA) analysis as directed by the manufacturer. In this study, human circRNA microarray v1.0 (Arraystar Inc.) containing 5396 circular RNA probes was used. In brief, total RNA was treated with RNase R for linear RNA removal and circRNA enrichment. Upon amplification, the obtained circRNAs were submitted to transcription for fluorescent cRNA production by the random priming method. Then, the fluorescent cRNAs were hybridized onto the Arraystar Human circRNA Array. Subsequently, the arrays were scanned on Agilent G2505C Scanner. The Agilent Feature Extraction software was used for image import and raw data were extracted. Differentially expressed circRNAs between the two groups were evaluated by *t*-test, and *P*-value correction for False Discovery Rate (FDR) was carried out using the Benjamini-Hochberg (BH) procedure. An absolute fold change value ≥1.5 and FDR *P* < 0.05 was considered statistically significant. Quantile normalization of raw data and subsequent data processing were performed with the R software limma package (version 2.7.10).

### Quantitative real-time PCR analysis

The cDNAs were obtained by reverse transcription from total RNA with a PrimeScript™ RT kit (Takara Bio Inc., Japan). SYBR®Premix Ex Taq™ II (TaKaRa) was used for fluorescence quantitative real-time PCR (RT-qPCR), with GAPDH as an internal control (Yin et al., 2017; Zhao et al., 2017). Divergent primers (Supplementary Table 1) were designed through the Circinteractome Divergent Primers web, verified via primer-BLAST, and synthesized by Shanghai Shenggong (Shanghai, China). RT-qPCR was performed on a ABI 7500 Real Time PCR System (Applied Biosystems, USA). The amounts of circRNAs were derived by the 2^−ΔΔ*Ct*^ method.

### Statistical analysis

Quantile normalization and subsequent data processing were performed in R. SPSS 17.0 (SPSS Inc.) was utilized for other statistics. Data are mean ± standard deviation (SD); normality was assessed by the Kolmogorov-Smirnov method. Student's *t*-test and Mann-Whitney *U*-test were employed to compare normally distributed parameters and those with skewed distribution, respectively. A receiver operating characteristic (ROC) curve was generated to evaluate the diagnostic value of circRNAs. The Spearman method was used for correlation analysis. The validated plasma biomarkers were entered into binary logistic regression models, and model selection was performed to determine the final combinations of biomarkers. *P* < 0.05 was considered statistically significant.

## Results

### Characteristics of the study population

We first analyzed plasma circRNAs of 3 patients with active pulmonary TB and 3 healthy control individuals by circRNA microarrays. In the validation stage, 170 pulmonary TB patients, 40 pneumonia patients, 40 COPD patients, 40 lung cancer patients, and 150 healthy controls were recruited. The clinical features of all participants are presented in Table 1.

**Table 1:** Characteristics of study subjects in the screening and validation stage.

**Characteristic**	**Microarray analysis**		**Validation**				
	**TB**	**Control**	**TB**	**Control**	**Lung cancer**	**Pneumonia**	**COPD**
Enrollment time	May, 2015	May, 2015	June, 2015 to Jan, 2017	June, 2015 to Jan, 2017	July, 2015 to Dec, 2016	July, 2015 to Dec, 2016	July, 2015 to Dec, 2016
Total number	3	3	170	150	40	40	40
Gender (male/female)	2/1	2/1	115/55	98/52	30/10	29/11	28/12
Age (years, mean)	37.5 ± 5.4	35.8 ± 6.9	44.5 ± 15.8	42.1 ± 13.3	49.9 ± 11.0	46.1 ± 14.2	48.7 ± 16.4
Smoking (Yes/no)	1/2	1/2	92/78	85/65	30/10	27/13	25/15
BCG vaccination (yes/no)	3/0	3/0	162/8	143/7	40/0	39/1	37/3
TST (Positive/Negative)	3/0	0/3	170/0	NA	NA	NA	NA
**SIGN AND SYMPTOMS (n)**							
Productive or unproductive cough	3	–	141	–	–	–	–
Weight loss	3	–	134	–	–	–	–
Fever	3	–	118	–	–	–	–
**SMEAR (n)**							
+	1	NA	90	NA	NA	NA	NA
++	1	NA	28	NA	NA	NA	NA
+++	1	NA	21	NA	NA	NA	NA
Negative	0	NA	31	NA	NA	NA	NA
**STATUS OF CHEST RADIOGRAPH (n)**							
Minimal	1	NA	79	NA	NA	NA	NA
Moderate	1	NA	56	NA	NA	NA	NA
Advanced	1	NA	35	NA	NA	NA	NA

*NA, not applicable*.

### Screening of differentially expressed circRNAs

**Figure 1:**
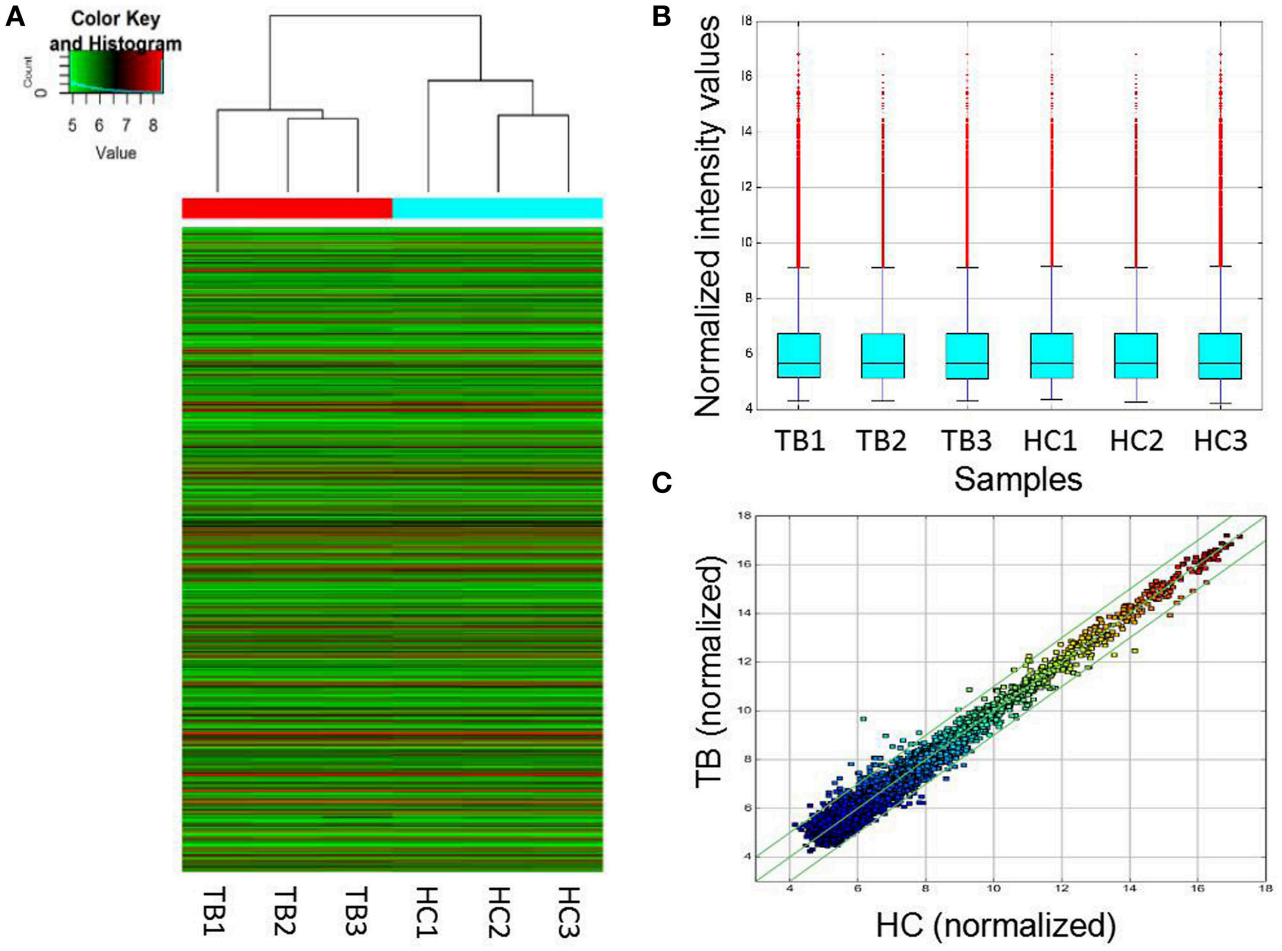
Differentially expressed circRNAs in TB patients (*n* = 3) and control individuals (*n* = 3). **(A)** Hierarchical clustering of circRNA expression profiles in the TB and control groups. “Red” and “green” indicate high and low relative expression levels, respectively. **(B)** Box plots showing the distributions of circRNAs in the two groups, which were similar after normalization. **(C)** In the scatter plot, circRNAs above the top and bottom green lines indicated more than 1.5-fold change in circRNA levels between the two groups. HC, healthy controls; TB, tuberculosis.

To assess whether circRNAs are differentially expressed between TB patients and normal subjects, plasma total RNA was extracted from 3 patients with active pulmonary TB and 3 age- and sex-matched healthy controls for circRNA microarray analysis. The box plots in Figure 1 show the normalized intensities for the plasma samples from TB patients and healthy controls. Hierarchical clustering and scatter plots revealed group differences. Sixty-one circRNAs showed differential expression levels between TB patients and controls, including 43 and 18 that were upregulated and downregulated in TB, respectively (fold change ≥1.5; *P* < 0.05) (Supplementary Table 2). The 15 most up-regulated and down-regulated circRNAs are presented in Table 2. The upregulated circRNAs included 36 exonic, 3 intronic, 3 sense overlapping and 1 antisense. Meanwhile, 14 exonic, 2 intronic, and 2 sense overlapping circRNAs were downregulated. To identify the most clinically applicable biomarkers, the 6 circRNAs showing the highest fold changes were selected from the upregulation group for further analysis: hsa_circ_0009024, hsa_circ_0001953, hsa_circ_0008297, hsa_circ_0003528, hsa_circ_0003524 and hsa_circ_0015879.

**Table 2:** Top 30 differently expressed circRNAs in TB patients.

**CircRNA ID**	***P*-value**	**FDR**	**FC**	**Regulation**	**CircRNA_type**	**Strand**	**Best_transcript**	**GeneSymbol**
hsa_circ_0009024	4.17569E-06	0.002492284	5.32391	Up	Exonic	+	NR_045128	TXLNGY
hsa_circ_0001953	5.48752E-05	0.004765914	4.66648	Up	Exonic	+	NM_003411	ZFY
hsa_circ_0008297	0.000235706	0.007462334	4.25936	Up	Exonic	+	NM_004660	DDX3Y
hsa_circ_0003528	0.008851434	0.04811339	3.71021	Up	Exonic	+	NM_021982	SEC24A
hsa_circ_0003524	0.000648282	0.011824622	3.63670	Up	Exonic	–	NM_001009993	FAM168B
hsa_circ_0015879	0.003051585	0.025521368	3.48609	Up	Exonic	+	NM_000299	PKP1
hsa_circ_0005232	0.000732236	0.012499195	2.97233	Up	Exonic	–	ENST00000403092	SLC8A1
hsa_circ_0044234	6.90949E-05	0.005050619	2.88108	Up	Exonic	–	NM_001256	CDC27
hsa_circ_0079262	0.002185264	0.021504814	2.59964	Up	Exonic	–	NM_024963	FBXL18
hsa_circ_0000957	0.003127342	0.025825725	2.59498	Up	Antisense	+	NM_017607	PPP1R12C
hsa_circ_0006903	0.000572627	0.011232802	2.49779	Up	Exonic	–	NM_015114	ANKLE2
hsa_circ_0059880	0.003497462	0.027589529	2.45421	Up	Exonic	+	NM_032819	ZNF341
hsa_circ_0051907	0.002823067	0.024494929	2.32641	Up	Sense overlapping	+	NM_001015	RPS11
hsa_circ_0070739	0.008585132	0.047220225	2.29498	Up	Exonic	+	NR_109983	LOC729218
hsa_circ_0006517	1.87953E-05	0.003817991	2.21372	Up	Exonic	+	NM_005578	LPP
hsa_circ_0002473	2.30534E-06	0.002191928	3.54805	Down	Exonic	+	NM_006260	DNAJC3
hsa_circ_0089837	0.000186077	0.006946271	3.21325	Down	Exonic	–	uc004crl.3	AK000470
hsa_circ_0014186	3.57663E-05	0.004521709	3.00623	Down	Exonic	+	NM_030918	SNX27
hsa_circ_0003641	3.86525E-05	0.004540802	2.90244	Down	Exonic	+	NM_000051	ATM
hsa_circ_0001490	0.000018765	0.003817991	2.89995	Down	Exonic	+	NM_004520	KIF2A
hsa_circ_0079385	2.18594E-05	0.003841024	2.67505	Down	Exonic	+	NM_018106	ZDHHC4
hsa_circ_0053944	0.000673145	0.0120992	2.66694	Down	Exonic	–	uc002roz.1	FAM98A
hsa_circ_0048764	0.000885593	0.013511581	2.45421	Down	Exonic	+	NM_015414	RPL36
hsa_circ_0092360	7.09269E-05	0.005055373	2.20000	Down	Intronic	+	ENST00000422514	RPL23A
hsa_circ_0003748	0.000163961	0.006608264	2.11369	Down	Exonic	–	NM_016291	IP6K2
hsa_circ_0000745	0.000339836	0.008957861	2.02089	Down	Exonic	+	NM_152904	SPECC1
hsa_circ_0000601	0.001165123	0.015424823	1.74041	Down	Exonic	–	NM_017672	TRPM7
hsa_circ_0000169	0.000178628	0.006792563	1.73952	Down	Sense overlapping	+	NM_133494	NEK7
hsa_circ_0002228	2.30882E-05	0.003841024	1.62324	Down	Exonic	–	NM_001101426	ISPD
hsa_circ_0092283	0.001758672	0.019370551	1.60165	Down	Intronic	–	ENST00000216181	MYH9

*CircRNA ID, The circRNA ID is in circBase (http://www.circbase.org/)*.

### Validation of circRNAs expression

**Figure 2:**
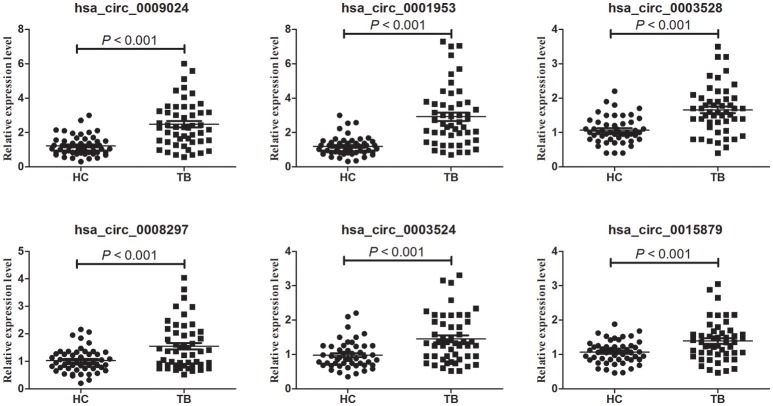
Validation of circRNA microarray profiles by RT-qPCR. The expression levels of six circRNAs were validated by RT-qPCR in plasma specimens from an independent cohort consisting of 50 TB patients and 50 healthy controls. The relative expression levels of circRNAs were normalized to GAPDH. Data are mean ± SD. Statistical analysis was performed by the nonparametric Mann-Whitney *U* test.

Microarray data were validated by RT-qPCR analysis of hsa_circ_0009024, hsa_circ_0001953, hsa_circ_0008297, hsa_circ_0003528, hsa_circ_0003524 and hsa_circ_0015879, in an independent cohort of 50 TB patients and 50 normal subjects. A good consistency between microarray and RT-qPCR data was obtained (Figure 2), as the levels of these 6 circRNAs were significantly increased in the TB patients.

### ROC curve analysis of differentially expressed circRNAs

**Figure 3:**
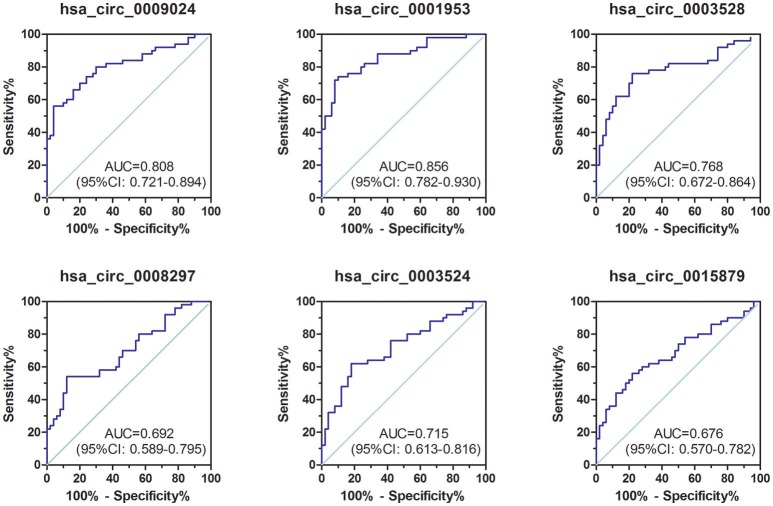
ROC curve analysis of confirmed circRNAs in plasma samples from TB patients in the validation set. TB group, *n* = 50; HC group, *n* = 50. AUC values are provided on the graphs.

To assess the diagnostic value of these confirmed plasma circRNAs as candidate biomarkers of TB, we performed ROC curve analysis. As shown in Figure 3, AUC values were larger than 0.500 for all 6 candidate circRNAs, suggesting their potential diagnostic value. Notably, the AUC for hsa_circ_0001953 reached 0.856 ([0.782–0.930], *P* < 0.001), and was the largest among the 6 circRNAs. The other AUC values were 0.808 ([0.721–0.894], *P* < 0.001) for hsa_circ_0009024, 0.768 ([0.672–0.864], *P* < 0.001) for hsa_circ_0003528, 0.715 ([0.613–0.816], *P* < 0.001) for hsa_circ_0003524, 0.692 ([0.589–0.795], *P* < 0.001) for hsa_circ_0008297 and 0.676 ([0.570–0.782], *P* = 0.002) for hsa_circ_0015879. Sensitivities and specificities of all circRNAs were obtained based on respective cut-off values (Table 3).

**Table 3:** Sensitivity and specificity of the candidate biomarkers in controls and TB patients.

**Candidate biomarkers**	**AUC (95% CI)**	**Sensitivity (%) (95% CI)**	**Specificity (%) (95% CI)**	***P*-value**	**Cut-off value**
hsa_circ_0009024	0.808 (0.721–0.894)	70.00 (55.39–82.14)	80.00 (66.28–89.97)	< 0.0001	1.625
hsa_circ_0001953	0.856 (0.782–0.930)	74.00 (59.65–85.37)	90.00 (78.19–96.67)	< 0.0001	1.760
hsa_circ_0003528	0.768 (0.672–0.864)	76.00 (61.83–86.94)	74.00 (59.65–85.37)	< 0.0001	1.275
hsa_circ_0008297	0.692 (0.589–0.795)	50.00 (35.53–74.47)	88.00 (75.69–95.47)	0.0009	1.365
hsa_circ_0003524	0.715 (0.613–0.816)	62.00 (47.18–75.35)	78.00 (64.04–88.47)	0.0002	1.245
hsa_circ_0015879	0.676 (0.570–0.782)	52.00 (37.42–66.34)	80.00 (66.28–89.97)	0.0024	1.335

### Plasma circRNA amounts are associated with the severity of TB

**Figure 4:**
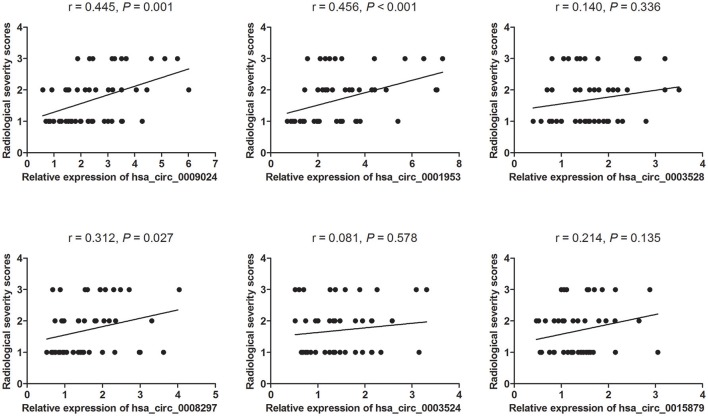
Associations of circRNA levels with lung injury in TB patients. Lung injury in TB patients was classified in a double-blind manner into three grades. Images are representative of minimal (1) (*n* = 25), moderate (2) (*n* = 15), and advanced (3) (*n* = 10) disease stages. The levels of six circRNAs were associated with the degree of lung injury in patients with active TB as assessed by the Spearman's rank correlation test. *P*- and *r*-values are specified in each chart.

TB cases were grouped in 3 groups as specified in Materials and Methods (minimal, moderate, and advanced disease). Then, associations of circRNA levels with the radiological score (severity index) were determined by the Spearman's rank correlation test. As depicted in Figure 4, 3 of the 6 circRNAs showed significant associations with the radiological score. Among these circRNAs, hsa_circ_0009024 and hsa_circ_0001953 were moderately correlated with disease severity, while hsa_circ_0008297 showed a weak correlation. Meanwhile, hsa_circ_0003528, hsa_circ_0003524 and hsa_circ_0015879 showed no associations with the radiological score. Considering the above findings, hsa_circ_0001953 and hsa_circ_0009024 were selected as potential biomarkers for TB diagnosis.

### Clinical confirmation of TB biomarkers

**Figure 5:**
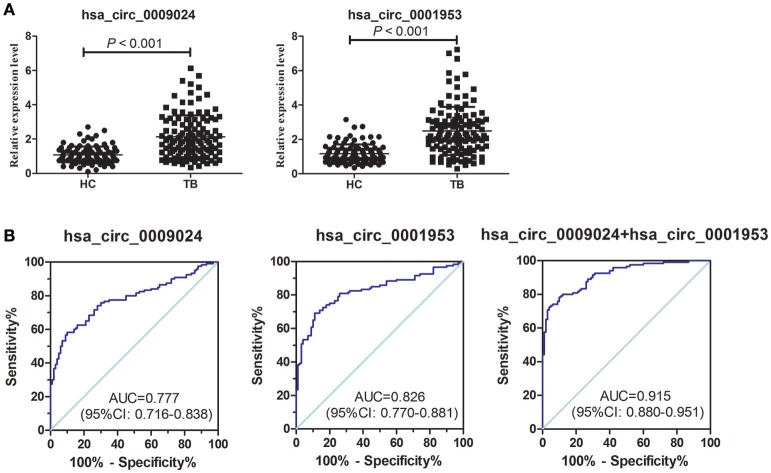
ROC curve analysis of hsa_circ_0009024 combined with hsa_circ_0001953. **(A)** Expression levels of hsa_circ_0009024 and hsa_circ_0001953 in TB patients (*n* = 120) and controls (*n* = 100). **(B)** ROC curves of hsa_circ_0009024 and hsa_circ_0001953 for discriminating between active TB patients and healthy controls. AUC values are given on the graphs.

The diagnostic potential of hsa_circ_0001953 and hsa_circ_0009024 in the clinical setting was determined by testing these circRNAs in a completely different sample comprising 120 TB and 100 healthy control subjects. Compared with healthy controls, the plasma levels of hsa_circ_0001953 and hsa_circ_0009024 were significantly higher in TB patients (Figure 5A, Supplementary Table 3). The AUC for hsa_circ_0001953 was 0.826 ([0.770–0.881], *P* < 0.001), indicating sensitivity and specificity of 69.17 and 89.00%, respectively. For hsa_circ_0009024, an AUC of 0.777 ([0.716–0.838], *P* < 0.001) was obtained; sensitivity and specificity were 60.00 and 86.00%, respectively. When hsa_circ_0001953 and hsa_circ_0009024 were combined, the AUC increased to 0.915 ([0.880–0.951], *P* < 0.001), with sensitivity and specificity of 72.50 and 96.00%, respectively (Figure 5B).

### Hsa_circ_0001953 and hsa_circ_0009024 expression levels in patients with TB, COPD, pneumonia, and lung cancer

**Figure 6:**
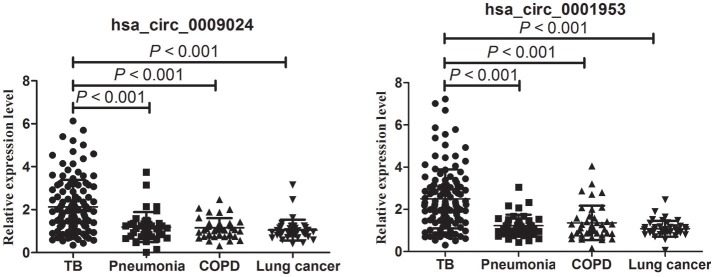
RT-qPCR assay validation of hsa_circ_0009024 and hsa_circ_0001953 expression levels in plasma specimens from 120 TB patients vs. 40 pneumonia, 40 COPD and 40 lung cancer patients, respectively. One-way ANOVA was used for statistical analysis.

As shown in Figure 6, hsa_circ_0001953 and hsa_circ_0009024 amounts were markedly increased in the TB group in comparison with COPD, pneumonia and lung cancer patients (all *P* < 0.001), respectively; however, the COPD, pneumonia, and lung cancer groups showed similar values (*P* > 0.05).

**Figure 7:**
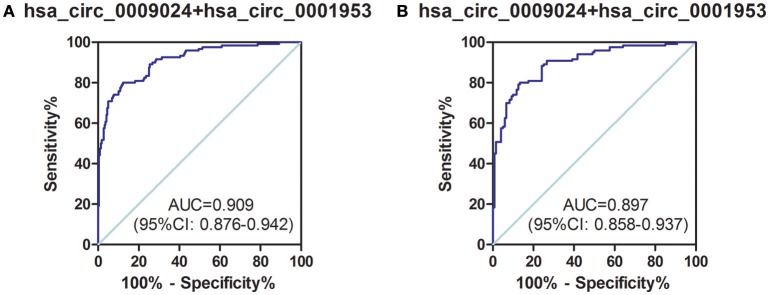
ROC curve analysis of hsa_circ_0009024 combined with hsa_circ_0001953 for determining risk-scores in **(A)** TB patients in the validation set vs. all controls (healthy controls in the validation set, as well as pneumonia, COPD, and lung cancer patients), **(B)** TB patients in the validation set vs. pneumonia, COPD, and lung cancer patients. AUC values are given on the graphs.

Next, a risk score based on hsa_circ_0001953 combined with hsa_circ_0009024 from the clinical validation set was further assessed in TB patients and all controls (healthy controls in the validation set, as well as COPD, pneumonia and lung cancer patients). The AUC for the latter risk score was 0.909 ([0.876–0.942], *P* < 0.001), with sensitivity and specificity of 80.00 and 87.73%, respectively (Figure 7A). This risk score also significantly discriminated the patients with TB from diseased controls (COPD, pneumonia and lung cancer patients), with an AUC of 0.897 ([0.858–0.937], *P* < 0.001); sensitivity and specificity were 79.17 and 86.67%, respectively (Figure 7B).

### Hsa_circ_0001953 and hsa_circ_0009024 expression levels are markedly decreased in TB patients upon successful therapy

**Figure 8:**
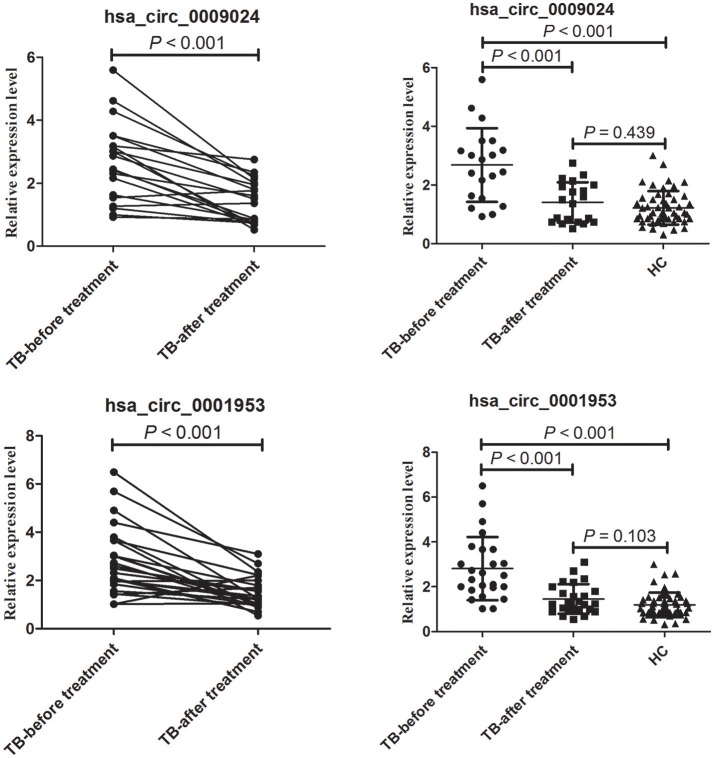
Changes in hsa_circ_0009024 and hsa_circ_0001953 levels in response to therapy. The levels of hsa_circ_0009024 and hsa_circ_0001953 were assessed by RT-qPCR. The dot plot shows relative levels of hsa_circ_0009024 and hsa_circ_0001953 in the same patients before and after treatment completion (*n* = 25). The relative levels of hsa_circ_0009024 and hsa_circ_0001953 in healthy controls (*n* = 50), pulmonary TB patients before (*n* = 25) and after treatment (*n* = 25) were plotted. Healthy controls and treated TB patients showed similar values. Statistical analysis was performed by Student's *t*-test.

In this study, hsa_circ_0001953 and hsa_circ_0009024 amounts were assessed in 25 TB cases pre- and post-treatment. In comparison with pre-treatment amounts, hsa_circ_0001953 and hsa_circ_0009024 showed lower values upon anti-TB therapy (Figure 8). Indeed, average hsa_circ_0001953 and hsa_circ_0009024 amounts returned almost to control values upon treatment, with the control and treated TB patients showing similar values (*P* > 0.05).

**Figure 9:**
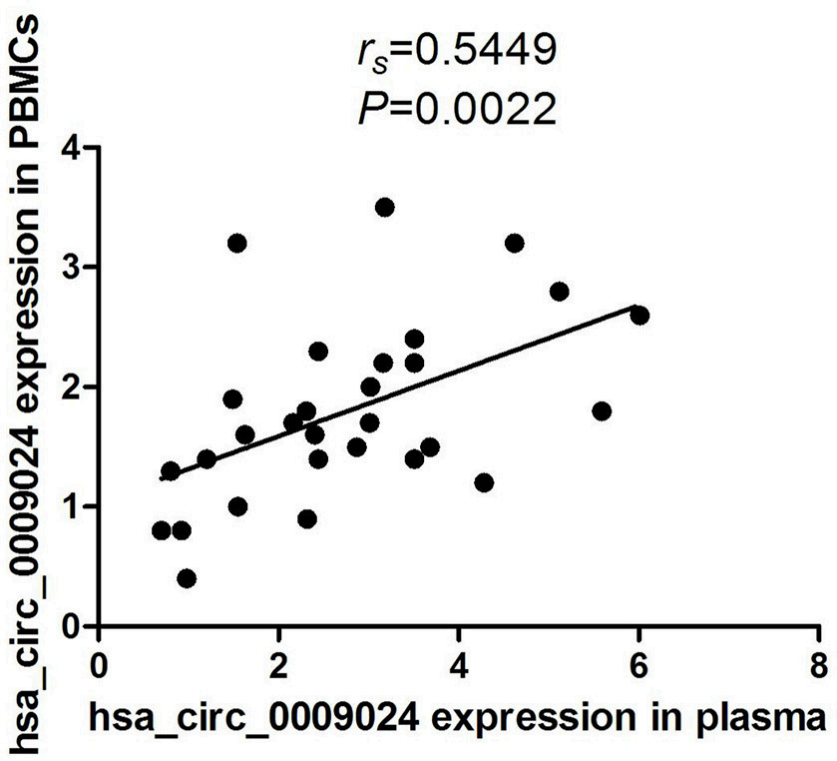
Spearman's rank correlation scatter plot of hsa_circ_0009024 levels in PBMCs samples and plasma.

Moreover, in our previous studies, we found that hsa_circ_0009024 was also significantly elevated in PBMCs from TB patients (Huang et al., 2018). We conducted Spearmen correlation analysis to explore the correlation between levels of hsa_circ_0009024 expression in plasma and in the corresponding PBMCs of the same patients. A moderate correlation was observed for hsa_circ_0009024 (*r*_*s*_ = 0.5449, *P* = 0.0022; Figure 9).

### CircRNA/miRNA interaction analysis

**Figure 10:**
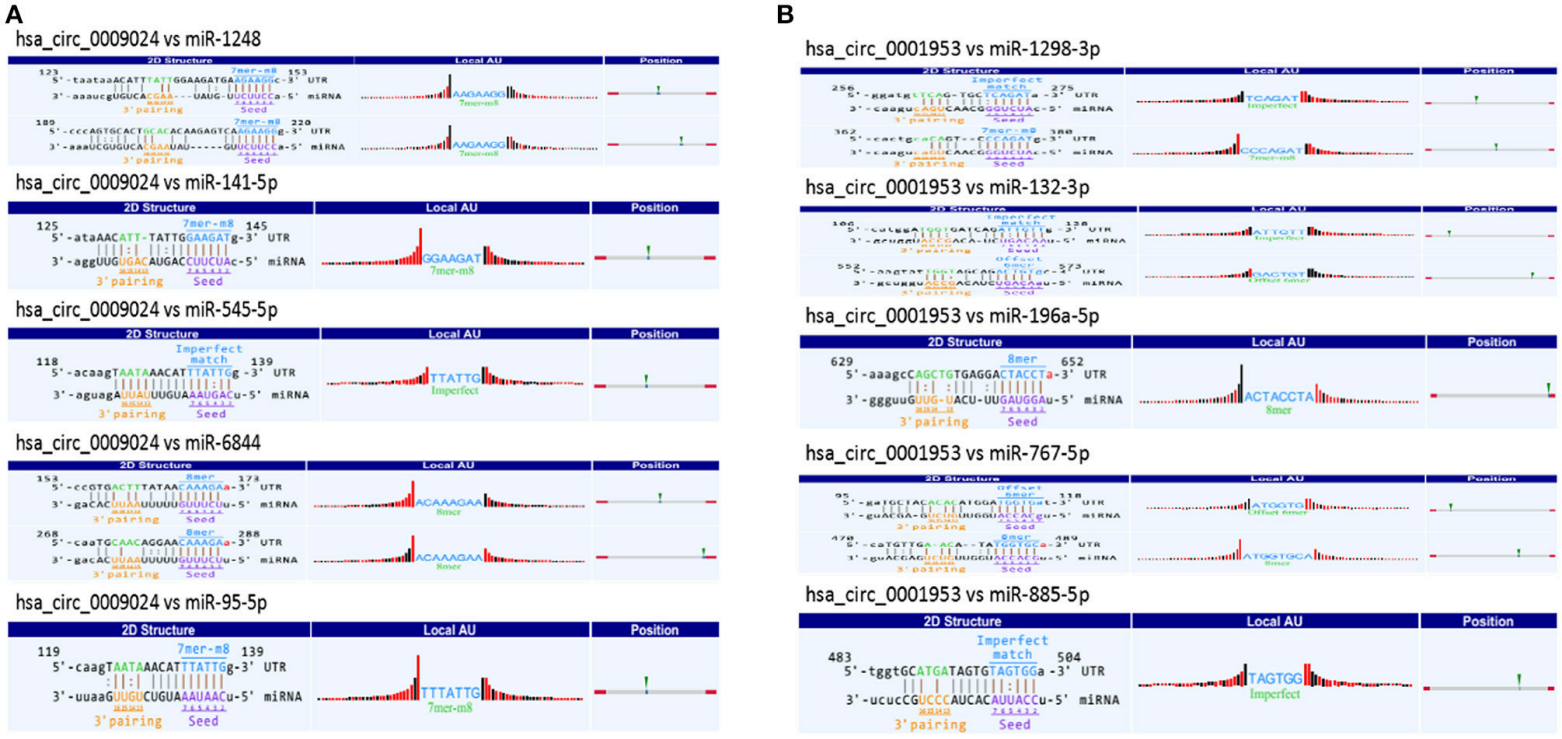
A snippet of the detailed annotation for circRNA/miRNA interaction. **(A)** hsa_circ_0009024. **(B)** hsa_circ_0001953. The circRNA/miRNA interaction was predicted with Arraystar's home-made miRNA target prediction software. Binding sites of conserved miRNAs with good mirSVR scores are represented.

CircRNAs may function as miRNA sponge to bind miRNAs and regulate expression of target genes. To evaluate the functions of hsa_circ_0001953 and hsa_circ_0009024, potential miRNA targets of the circRNAs were searched using the Arraystar's home-made miRNA target prediction software. The details of the molecular interactions between these two circRNAs and above target miRNAs are depicted in Figure 10.

## Discussion

CircRNAs are newly discovered endogenous non-coding RNAs featuring structural stability, high abundance, and tissue-specificity. To date, studies have revealed the promising role of circRNAs as biomarkers of tumors (He et al., 2017), cardiovascular disease (Fan et al., 2017), Alzheimer's disease (Lukiw, 2013), and other diseases (Chen et al., 2016; Tang et al., 2017). Recently, Qian et al. identified seven circRNAs (hsa_circ_0000414, hsa_circ_0000681, hsa_circ_0002113, hsa_circ_0002362, hsa_circ_0002908, hsa_circ_0008797, hsa_circ_0063179) have the potential to serve as novel potential biomarkers for diagnosis of active TB (Qian et al., 2018). Zhuang et al. showed that hsa_circ_0005836 in PBMCs might serve as a novel potential biomarker of TB infection (Zhuang et al., 2017). Our previous study revealed that several PBMC circRNAs can be used as potential biomarkers for TB (Huang et al., 2017, 2018). However, due to the complexity of RNA extraction from PBMCs and challenging quality control, it is difficult to meet the needs for clinical application. Here, circRNA amounts in plasma specimens from active TB patients and healthy subjects were assessed by circRNA microarray.

As shown above, 43 upregulated and 18 downregulated circRNAs were found by microarrays in the TB group. To identify clinically relevant biomarkers, 6 significantly overexpressed circRNAs were identified, and associations of their amounts with TB severity were evaluated. The validation study demonstrated that plasma amounts of these 6 circRNAs (hsa_circ_0009024, hsa_circ_0001953, hsa_circ_0008297, hsa_circ_0003528, hsa_circ_0003524 and hsa_circ_0015879) were remarkably increased in the TB patients analyzed. ROC curve analysis suggested that hsa_circ_0001953 had a significant value for TB diagnosis, followed by hsa_circ_0009024, hsa_circ_0003528, hsa_circ_0003524, hsa_circ_0008297, and hsa_circ_0015879. Furthermore, Spearman's rank correlation analysis revealed that hsa_circ_0001953 and hsa_circ_0009024 amounts were moderately correlated with the radiological score, implying that hsa_circ_0001953 and hsa_circ_0009024 might be involved in TB pathology.

To determine whether hsa_circ_0001953 and hsa_circ_0009024 could constitute diagnostic biomarkers of TB, they were assessed in larger patient cohorts. Based on ROC curves, cut-off values that best differentiated TB patients from healthy individuals were selected. Hsa_circ_0001953 had a sensitivity of 69.17% and a specificity of 89.00% for TB, while hsa_circ_0009024 had sensitivity and specificity of 60.00 and 86.00%, respectively. Hsa_circ_0001953 demonstrated a higher discriminating ability compared with hsa_circ_0009024, although not statistically significant (*P* > 0.05). ROC analysis using both targets in combination yielded an increased AUC of 0.915, with 72.50% sensitivity and 96.00% specificity in discriminating TB patients from normal controls, indicating additive effects in diagnostic potential of the two circRNAs. These diagnostic abilities were quite comparable to those reported for blood biomarkers of TB, especially in terms of specificity (Fu et al., 2011; Miotto et al., 2013; Zhang et al., 2013; Latorre et al., 2015). Next, the ability of the combination to effectively segregate TB from other lung ailments (COPD, pneumonia, and lung cancer) was assessed. As shown above, hsa_circ_0001953 and hsa_circ_0009024 might constitute TB signature circRNAs and represent potential TB biomarkers. Moreover, the levels of hsa_circ_0001953 and hsa_circ_0009024 in TB patients returned to normal after effective therapy.

In one of our previous study, we found that the levels of hsa_circ_0009024 were also significantly elevated in PBMCs from TB patients compared to those of healthy control (Huang et al., 2018). In that study we detected the levels of circRNAs only in PMBCs, but not in plasma. While the subjects recruited in these two studies were not the same, there were 52 TB patients and 21 healthy controls overlapped. Thus, the levels of hsa_circ_0009024 were detected in both PBMCs and plasma in these overlapped subjects. Combining the results of these two studies we found that the levels of hsa_circ_0009024 in plasma were moderately correlated with those in PBMCs. Although further confirmation needed these results suggest that PBMCs may secrete circRNAs to the plasma. In conformity with our findings, Li et al. recently reported that hsa_circ_0001017 and hsa_circ_0061276 can be secreted from gastric cancer cells (Li et al., 2017). However, the level of another circRNA, hsa_circ_0001953, which was found to increase significantly in plasma, had no significant difference in the PBMCs between TB patients and healthy controls (Huang et al., 2018), which suggested that other cells may also secret circRNAs into plasma.

It is known circRNAs might function as miRNA sponges or regulate parent genes to alter the disease course (Ebbesen et al., 2016; Greene et al., 2017). Multiple miRNAs regulate immune responses to infection by mycobacteria (Abdalla et al., 2016; Bettencourt et al., 2016; Sabir et al., 2018). For instance, miR-125b reduces TNF amounts via direct binding to its 3′UTR (Rajaram et al., 2011), whereas miR-21 suppresses IL-12 synthesis via IL-12p35 inhibition, impairing responses to Mycobacterium (Wu et al., 2012). *Mycobacterium*-induced miR-155 targets Rheb to enhance autophagy in macrophages, which in turn promote the elimination of intracellular bacilli (Wang et al., 2013). Iwai et al. found miRNA-155 knockout mice show increased susceptibility to *M. tuberculosis* compared with wild-type animals (Iwai et al., 2015). Bioinformatics predicted that miR-767-5p, miR-1298-3p, miR-132-3p, miR-885-5p, and miR-196a-5p might be potential targets of hsa_circ_0001953. Hsa_circ_0009024 was shown to potentially bind miR-6844, miR-1248, miR-141-5p, miR-545-5p, and miR-95-5p. A previous study demonstrated that *M. tuberculosis* decreases human macrophage IFN-γ responsiveness through miR-132 (Ni et al., 2014). However, due to the limited knowledge of miRNA and circRNA properties, the potential functions of the circRNA-miRNA axis in the pathogenesis of TB require further investigation.

However, the current study had many limitations. First, because of the policy of universal BCG vaccination in China, it is very challenging to recruit control subjects with negative TST. Meanwhile, because IGRA has not been popularized in China due to its high cost (~110 USD/test), we did not assess control subjects for latent TB in this study. In addition, the subjects were limited to the Chinese Han population, and only 25 TB patients completed follow-up. Therefore, the current findings require confirmation in larger and more diverse samples.

In addition, a fact we cannot avoid is that as a new biomarker for disease diagnosis, the consistency or reproducibility of circRNA in different studies is not satisfactory. Due to the fact that the expression of circRNA is influenced by many factors, such as the specimen type, detection technology and individual difference, the candidate circRNAs obtained in different laboratories are often different, even targeting the same disease. We noticed that the circRNAs with potential to serve as biomarker for TB diagnosis in this study (hsa_circ_0001953 and hsa_circ_0009024) are different from the candidate circRNAs obtained by Qian et al. (2018). By comparing these two studies, we speculate that the inconsistent result might result from four main differences between these two studies. First, the specimen we studied is plasma, not PBMCs in Qian's study (Qian et al., 2018). Second, we screened the differentially expressed circRNAs by circRNA expression microarray, while Qian et al. characterized the circRNA expression profile firstly by using RNA sequencing, followed by circRNA expression microarrays. As we know, the data obtained by RNA sequencing is far bigger and more complex than circRNA expression microarrays. Third, the differentially expressed circRNA was screened with criterion of >1.5-fold change and a *P*-value < 0.05, which was more harsh than the criterion in Qian's study. A *P*-value < 0.05 were deemed differentially expressed in Qian's study (Qian et al., 2018). Finally, the differentially expressed circRNAs chosen for validation were restricted in 5 given KEGG pathways, those were “Cytokine-cytokine receptor interaction,” “Chemokine signaling pathway,” “Fc gamma R-mediated phagocytosis,” “Neurotrophin signaling pathway,” and “Bacterial invasion of epithelial cells.” However, the 6 circRNAs showing the highest fold changes were chosen from the up-regulation group for validation and further analysis in this study.

Overall, differentially expressed circRNAs were detected in plasma specimens from active TB and normal control patients. Our data indicated that hsa_circ_0001953 and hsa_circ_0009024 levels in plasma could constitute new diagnostic biomarkers for TB diagnosis. Comprehensive studies are warranted to unveil the mechanisms by which circRNAs affect TB infection. And, considering the inconsistency of the findings between different laboratories, a more rigorous, large sample and multicenter study is needed to further confirm the reliability and reproducibility of these circRNAs in TB diagnosis.

## Author contributions

ZH, QL, and JL designed the study. RS, CQ, and YP collected clinical specimens. ZH, RS, and CQ performed laboratory assays. ZH, QL, and JL performed data analysis. All authors read and approved the final manuscript.

### Conflict of interest statement

The authors declare that the research was conducted in the absence of any commercial or financial relationships that could be construed as a potential conflict of interest.

## Abbreviations

TB: tuberculosis
circRNAs: (circRNAs)
AFB: acid-fast bacilli
PBMCs: peripheral blood mononuclear cells
ceRNAs: competing endogenous RNAs
miRNAs: microRNAs
FDR: false discovery rate
SD: standard deviation
ROC: receiver operating characteristic
AUC: area under curve
RT-qPCR, quantitative real-time polymerase chain reaction, GAPDH: glyceraldehyde-3-phosphate dehydrogenase
COPD: chronic obstructive pulmonary disease
BCG: Bacille Calmette-Guérin
TST: tuberculin skin test
NA: not applicable
IGRA: Interferon gamma release assay.

## References

[B1] Abakay O. Abakay A. Sen H. S. Tanrikulu A. C. (2015). The relationship between inflammatory marker levels and pulmonary tuberculosis severity. Inflammation 38, 691–696. 10.1007/s10753-014-9978-y 25028104

[B2] Abdalla A. E. Duan X. Deng W. Zeng J. Xie J. (2016). MicroRNAs play big roles in modulating macrophages response toward mycobacteria infection. Infect. Genet. Evol. 45, 378–382. 10.1016/j.meegid.2016.09.023 27693402

[B3] Albert H. Nathavitharana R. R. Isaacs C. Pai M. Denkinger C. M. Boehme C. C. (2016). Development, roll-out and impact of Xpert MTB/RIF for tuberculosis: what lessons have we learnt and how can we do better? Eur. Respir. J. 48, 516–525. 10.1183/13993003.00543-2016 27418550 PMC4967565

[B4] Bettencourt P. Pires D. Anes E. (2016). Immunomodulating microRNAs of mycobacterial infections. Tuberculosis (Edinb). 97, 1–7. 10.1016/j.tube.2015.12.004 26980489

[B5] Chen Y. Li C. Tan C. Liu X. (2016). Circular RNAs: a new frontier in the study of human diseases. J. Med. Genet. 53, 359–365. 10.1136/jmedgenet-2016-103758 26945092

[B6] Dheda K. Barry C. E. III. Maartens G. (2016). Tuberculosis. Lancet 387, 1211–1226. 10.1016/S0140-6736(15)00151-8 26377143 PMC11268880

[B7] Ebbesen K. K. Hansen T. B. Kjems J. (2016). Insights into circular RNA biology. RNA Biol. 14, 1035–1045. 10.1080/15476286.2016.1271524 27982727 PMC5680708

[B8] Fan X. Weng X. Zhao Y. Chen W. Gan T. Xu D. (2017). Circular RNAs in cardiovascular disease: an overview. Biomed Res. Int. 2017:5135781 10.1155/2017/5135781 28210621 PMC5292166

[B9] Fu Y. Yi Z. Wu X. Li J. Xu F. (2011). Circulating microRNAs in patients with active pulmonary tuberculosis. J. Clin. Microbiol. 49, 4246–4251. 10.1128/JCM.05459-11 21998423 PMC3232949

[B10] García-Basteiro A. L. Ismail M. R. Carrilho C. Ussene E. Castillo P. Chitsungo D.. (2016). The role of Xpert MTB/RIF in diagnosing pulmonary tuberculosis in post-mortem tissues. Sci. Rep. 6:20703. 10.1038/srep20703 26860394 PMC4748254

[B11] Greene J. Baird A. M. Brady L. Lim M. Gray S. G. McDermott R.. (2017). Circular RNAs: biogenesis, function and role in human diseases. Front. Mol. Biosci. 4:38. 10.3389/fmolb.2017.00038 28634583 PMC5459888

[B12] He J. Xie Q. Xu H. Li J. Li Y. (2017). Circular RNAs and cancer. Cancer Lett. 396, 138–144. 10.1016/j.canlet.2017.03.027 28342987

[B13] Huang Z. Su R. Deng Z. Xu J. Peng Y. Luo Q.. (2017). Identification of differentially expressed circular RNAs in human monocyte derived macrophages response to Mycobacterium tuberculosis infection. Sci. Rep. 7:13673. 10.1038/s41598-017-13885-0 29057952 PMC5651861

[B14] Huang Z. K. Yao F. Y. Xu J. Q. Deng Z. Su R. G. Peng Y. P.. (2018). Microarray expression profile of circular RNAs in peripheral blood mononuclear cells from active tuberculosis patients. Cell. Physiol. Biochem. 45, 1230–1240. 10.1159/000487454 29448254

[B15] Iwai H. Funatogawa K. Matsumura K. Kato-Miyazawa M. Kirikae F. Kiga K.. (2015). MicroRNA-155 knockout mice are susceptible to Mycobacterium tuberculosis infection. Tuberculosis (Edinb). 95, 246–250. 10.1016/j.tube.2015.03.006 25846955

[B16] Kranzer K. Khan P. Godfrey-Fausset P. Ayles H. Lonnroth K. (2016). Tuberculosis control. Lancet 387, 1159–1160. 10.1016/S0140-6736(16)00710-8 27025331

[B17] Kulcheski F. R. Christoff A. P. Margis R. (2016). Circular RNAs are miRNA sponges and can be used as a new class of biomarker. J. Biotechnol. 238, 42–51. 10.1016/j.jbiotec.2016.09.011 27671698

[B18] Latorre I. Leidinger P. Backes C. Domínguez J. de Souza-Galvão M. L. Maldonado J.. (2015). A novel whole-blood miRNA signature for a rapid diagnosis of pulmonary tuberculosis. Eur. Respir. J. 45, 1173–1176. 10.1183/09031936.00221514 25657026

[B19] Li H. Li K. Lai W. Li X. Wang H. Yang J.. (2018). Comprehensive circular RNA profiles in plasma reveals that circular RNAs can be used as novel biomarkers for systemic lupus erythematosus. Clin. Chim. Acta 480, 17–25. 10.1016/j.cca.2018.01.026 29360436

[B20] Li T. Shao Y. Fu L. Xie Y. Zhu L. Sun W.. (2017). Plasma circular RNA profiling of patients with gastric cancer and their droplet digital RT-PCR detection. J. Mol. Med. (Berl). 96, 85–96. 10.1007/s00109-017-1600-y 29098316

[B21] Liu J. Liu T. Wang X. He A. (2017). Circles reshaping the RNA world: from waste to treasure. Mol. Cancer 16:58 10.1186/s12943-017-0630-y 28279183 PMC5345220

[B22] Lukiw W. J. (2013). Circular RNA (circRNA) in Alzheimer's disease (AD). Front. Genet. 4:307. 10.3389/fgene.2013.00307 24427167 PMC3875874

[B23] Memczak S. Papavasileiou P. Peters O. Rajewsky N. (2015). Identification and Characterization of Circular RNAs As a New Class of Putative Biomarkers in Human Blood. PLoS ONE 10:e0141214. 10.1371/journal.pone.0141214 26485708 PMC4617279

[B24] Miotto P. Mwangoka G. Valente I. C. Norbis L. Sotgiu G. Bosu R.. (2013). miRNA signatures in sera of patients with active pulmonary tuberculosis. PLoS ONE 8:e80149. 10.1371/journal.pone.0080149 24278252 PMC3836984

[B25] Ng W. L. Marinov G. K. Chin Y. M. Lim Y. Y. Ea C. K. (2017). Transcriptomic analysis of the role of RasGEF1B circular RNA in the TLR4/LPS pathway. Sci. Rep. 7:12227. 10.1038/s41598-017-12550-w 28947785 PMC5612941

[B26] Ni B. Rajaram M. V. Lafuse W. P. Landes M. B. Schlesinger L. S. (2014). Mycobacterium tuberculosis decreases human macrophage IFN-gamma responsiveness through miR-132 and miR-26a. J. Immunol. 193, 4537–4547. 10.4049/jimmunol.1400124 25252958

[B27] Qian Z. Liu H. Li M. Shi J. Li N. Zhang Y.. (2018). Potential diagnostic power of blood circular RNA expression in active pulmonary tuberculosis. EBioMedicine 27, 18–26. 10.1016/j.ebiom.2017.12.007 29248507 PMC5828303

[B28] Rajaram M. V. Ni B. Morris J. D. Brooks M. N. Carlson T. K. Bakthavachalu B.. (2011). Mycobacterium tuberculosis lipomannan blocks TNF biosynthesis by regulating macrophage MAPK-activated protein kinase 2 (MK2) and microRNA miR-125b. Proc. Natl. Acad. Sci. U.S.A. 108:17408–17413. 10.1073/pnas.1112660108 21969554 PMC3198317

[B29] Rong D. Sun H. Li Z. Liu S. Dong C. Fu K.. (2017). An emerging function of circRNA-miRNAs-mRNA axis in human diseases. Oncotarget 8, 73271–73281. 10.18632/oncotarget.19154 29069868 PMC5641211

[B30] Sabir N. Hussain T. Shah S. Z. A. Peramo A. Zhao D. Zhou X. (2018). miRNAs in tuberculosis: new avenues for diagnosis and host-directed therapy. Front. Microbiol. 9:602. 10.3389/fmicb.2018.00602 29651283 PMC5885483

[B31] Tang C. M. Zhang M. Huang L. Hu Z. Q. Zhu J. N. Xiao Z.. (2017). CircRNA_000203 enhances the expression of fibrosis-associated genes by derepressing targets of miR-26b-5p, Col1a2 and CTGF, in cardiac fibroblasts. Sci. Rep. 7:40342. 10.1038/srep40342 28079129 PMC5228128

[B32] Wang J. Yang K. Zhou L. Minhaowu Wu Y. Zhu M.. (2013). MicroRNA-155 promotes autophagy to eliminate intracellular mycobacteria by targeting Rheb. PLoS Pathog. 9:e1003697. 10.1371/journal.ppat.1003697 24130493 PMC3795043

[B33] WHO (2017). Global Tuberculosis Report 2017. Geneva: WHO, Available online at: http://www.who.int/tb/publications/global_report/en/

[B34] Wu Z. Lu H. Sheng J. Li L. (2012). Inductive microRNA-21 impairs anti-mycobacterial responses by targeting IL-12 and Bcl-2. FEBS Lett. 586, 2459–2467. 10.1016/j.febslet.2012.06.004 22710123

[B35] Yin W. B. Yan M. G. Fang X. Guo J. J. Zhang R. P. Xiong W. (2017). Circulating circular RNA hsa_circ_0001785 acts as a diagnostic biomarker for breast cancer detection. Clin. Chim. Acta. 10.1016/j.cca.2017.10.011. [Epub ahead of print]. 29045858

[B36] Zhang X. Guo J. Fan S. Li Y. Wei L. Yang X.. (2013). Screening and identification of six serum microRNAs as novel potential combination biomarkers for pulmonary tuberculosis diagnosis. PLoS ONE 8:e81076. 10.1371/journal.pone.0081076 24349033 PMC3857778

[B37] Zhao K. Zhao Q. Guo Z. Chen Z. Hu Y. Su J.. (2018). Hsa_Circ_0001275: a potential novel diagnostic Biomarker for postmenopausal osteoporosis. Cell. Physiol. Biochem. 46, 2508–2516. 10.1159/000489657 29742503

[B38] Zhao Z. Li X. Gao C. Jian D. Hao P. Rao L.. (2017). Peripheral blood circular RNA hsa_circ_0124644 can be used as a diagnostic biomarker of coronary artery disease. Sci. Rep. 7:39918. 10.1038/srep39918 28045102 PMC5206672

[B39] Zhuang Z. G. Zhang J. A. Luo H. L. Liu G. B. Lu Y. B. Ge N. H.. (2017). The circular RNA of peripheral blood mononuclear cells: Hsa_circ_0005836 as a new diagnostic biomarker and therapeutic target of active pulmonary tuberculosis. Mol. Immunol. 90, 264–272. 10.1016/j.molimm.2017.08.008 28846924

