# Supplementary material for: Plasma Circular RNAs hsa_circ_0001953 and hsa_circ_0009024 as Diagnostic Biomarkers for Active Tuberculosis
Source: Front Microbiol. 2018 Aug 30;9:2010. doi: 10.3389/fmicb.2018.02010 (PMC6126419; doi:10.3389/fmicb.2018.02010)
Supplement: Supplementary file 1 [file Table_1.doc]

**Plasma circular RNAs hsa_circ_0001953 and hsa_circ_0009024 as diagnostic biomarkers for active tuberculosis**

Zikun Huang^1^, Rigu Su^1^, Cheng Qing^2^, Yiping Peng^3^, Qing Luo^1^, Junming Li^1^

^1^Department of Clinical Laboratory, the First Affiliated Hospital of Nanchang University, Nanchang 33006, China.

^2^Intensive Care Unit, the First Affiliated Hospital of Nanchang University, Nanchang, 330006, China.

^3^Department of Tuberculosis, Jiangxi Chest Hospital, Nanchang 330006, China.

| **Table S1.** Primers used for RT-qPCR analysis of circRNA and mRNA levels | | |
| --- | --- | --- |
| Name | Primer sequence 5’-3’ | Product size (bp) |
| GAPDH | F: TGTTGCCATCAATGACCCCTT | 202 |
|  | R: CTCCACGACGTACTCAGCG |  |
| hsa_circ_0009024 | F: TTCCTGAGCAAGAAGTAGCCC | 117 |
|  | R: TAACCCACAAAGTCCAGCTTCT |  |
| hsa_circ_0001953 | F: AGTCATAGATGCCAGCGGGA | 152 |
|  | R: GAGTTTGGCTCTTGTGGCTG |  |
| hsa_circ_0008297 | F: GTAGAGGCAACCGGCAGTAA | 128 |
|  | R: GCTGCAACTCCAACCTGAAC |  |
| hsa_circ_0003528 | F: GCTCTCCTTAAACAGGATATACACA | 113 |
|  | R: TGTCCACTGAGAAGGAATAAGTCA |  |
| hsa_circ_0003524 | F: CAGCAGCTCCTGCCTATTCT | 121 |
|  | R: GCATTTGCATAGGGAACCCC |  |
| hsa_circ_0015879 | F: CAGGAACTTCACCTCCCGATT | 125 |
|  | R: TGCTGGACGTTCTGGTTGGG |  |
